# Effects of di(*n*-butyl) phthalate exposure on foetal rat germ-cell number and differentiation: identification of age-specific windows of vulnerability

**DOI:** 10.1111/j.1365-2605.2010.01140.x

**Published:** 2011-10

**Authors:** M S Jobling, G R Hutchison, S van den Driesche, R M Sharpe

**Affiliations:** MRC Human Reproductive Sciences Unit, Centre for Reproductive Biology, The Queen's Medical Research InstituteEdinburgh, UK

**Keywords:** carcinoma-in-situ, DAZL, DMRT1, germ-cell aggregation, germ-cell number, OCT4, testicular germ-cell cancer, VASA

## Abstract

Environmental factors are implicated in increased incidence of human testicular germ-cell cancer (TGCC). TGCC has foetal origins and may be one component of a testicular dysgenesis syndrome (TDS). Certain phthalates induce TDS in rats, including effects on foetal germ cells (GC). As humans are widely exposed to phthalates, study of the effects of phthalates on foetal rat GC could provide an insight into the vulnerability of foetal GC to disruption by environmental factors, and thus to origins of TGCC. This study has therefore characterized foetal GC development in rats after in utero exposure to di(*n*-butyl) phthalate (DBP) with emphasis on GC numbers/proliferation, differentiation and time course for inducing effects. Pregnant rats were treated orally from embryonic day 13.5 (e13.5) with 500 mg/kg/day DBP for varying periods. GC number, proliferation, apoptosis, differentiation (loss of OCT4, DMRT1 expression, DMRT1 re-expression, GC migration) and aggregation were evaluated at various foetal and postnatal ages. DBP exposure reduced foetal GC number by ∼60% by e15.5 and prolonged GC proliferation, OCT4 and DMRT1 immunoexpression; these effects were induced in the period immediately after testis differentiation (e13.5–e15.5). In contrast, DBP-induced GC aggregation stemmed from late gestation effects (beyond e19.5). Foetal DBP exposure delayed postnatal resumption of GC proliferation, leading to bigger deficits in numbers, and delayed re-expression of DMRT1 and radial GC migration. Therefore, DBP differentially affects foetal GC in rats according to stage of gestation, effects that may be relevant to the human because of their nature (OCT4, DMRT1 effects) or because similar effects are demonstrable in vitro on human foetal testes (GC number). Identification of the mechanisms underlying these effects could give a new insight into environment-sensitive mechanisms in early foetal GC development that could potentially be relevant to TGCC origins.

## Introduction

Testicular germ-cell cancer (TGCC) is the most common cancer of young Caucasian men with a peak incidence occurring at around 25–30 years (Skakkebaek *et al.*, 2001). In most Western countries, TGCC has increased progressively in incidence over the last 40–70 years, indicative of lifestyle/environmental causes (Bray *et al.*, 2006). It is widely accepted that TGCC develops from pre-malignant carcinoma-in-situ (CIS) cells, which in turn result from foetal germ cells (GCs) that have failed to undergo normal differentiation in foetal/early postnatal life and have failed to switch off pluripotency characteristics (Rajpert-De Meyts, 2006; Van Casteren *et al.*, 2009). If CIS cells are identified in a testis, it predicts that TGCC will eventually develop (Skakkebaek *et al.*, 2001). Two major unresolved issues about CIS are: (1) why does differentiation fail in some foetal GC, and (2) what lifestyle/environmental factors can influence this, and thus explain the progressive increase in TGCC incidence.

TGCC is hypothesized to form part of a testicular dysgenesis syndrome (TDS), because of its foetal origins and its increased incidence in males with masculinization/reproductive developmental disorders (Skakkebaek *et al.*, 2001; Sharpe & Skakkebaek, 2008). An animal model for TDS, involving administration of certain phthalate esters, such as di(*n*-butyl) phthalate (DBP), to female rats during pregnancy has been established and results in a spectrum of disorders similar to TDS in humans (Gray *et al.*, 2000; Barlow & Foster, 2003; Fisher *et al.*, 2003), and some evidence points towards similar phthalate effects in humans (Swan *et al.*, 2005). Foetal exposure of rats to DBP, or to other phthalates that can induce a TDS-like syndrome, causes major effects on the foetal GC, including a reduction in numbers plus multinucleation and abnormal aggregation (Mylchreest *et al.*, 2000; Mylchreest *et al.*, 2002; Parks *et al.*, 2000; Fisher *et al.*, 2003; Ferrara *et al.*, 2006; Boekelheide *et al.*, 2009). Moreover, some of these phthalate effects have been recapitulated in vitro using foetal testis explants from the rat (Chauvigné*et al.*, 2009), mouse (Lehraiki *et al.*, 2009) and human (Lambrot *et al.*, 2009), demonstrating the potential relevance of the animal studies to the human.

Humans are widely exposed to phthalates (Silva *et al.*, 2004a), notably to DBP and diethylhexyl phthalate (DEHP), both of which cause similar effects on the foetal rat testis (Howdeshell *et al.*, 2008a). DBP/DEHP exposure of rats does not lead to formation of recognizable CIS cells or to development of TGCC in adulthood, but an earlier study showed that foetal exposure of rats to DBP resulted in delayed ‘differentiation’ of some foetal gonocytes (Ferrara *et al.*, 2006), an effect potentially relevant to CIS origins (Rajpert-De Meyts, 2006). From the evidence available, it is unclear if this ‘differentiation effect’ is unique and/or whether it is related to the other GC effects described. The objective of the present studies was to characterize the GC effects of DBP in rats, especially those relating to their numbers and differentiation, to identify the timing of phthalate-sensitive periods in foetal GC development, as these could lead to identification of early foetal GC mechanisms that are vulnerable to disruption by environmental factors, which might then shed light on CIS origins.

## Materials and methods

### Animal welfare, treatments, sample collection and processing

Wistar rats were maintained under United Kingdom Home Office guidelines in our own animal facility and fed a soy-free breeding diet (SDS, Dundee, Scotland). Experiments were conducted under Project Licence approval from the UK Home Office, which includes an ethical review step.

Time-mated females were subjected to daily oral gavage with either 500 mg/kg DBP (Sigma-Aldrich Co. Ltd., Dorset, UK) in 1 mL/kg corn oil or with the vehicle (control); the DBP was 99% pure according to the supplier. For the main experiments involving foetal recovery of tissues (e14.4–e21.5), treatments were administered daily until 24 h before the dam was killed; where recovery of tissues was postnatal, dams were treated daily from e13.5 to e21.5. In some studies, DBP or vehicle was administered from e13.5 to e15.5 (termed ‘early window’) and exposed animals subsequently killed on e17.5, e19.5 or e21.5. Similarly, in one experiment, DBP/vehicle treatment was confined to e19.5–e20.5 (‘late window’) and control and treated animals were then killed on e21.5. In all studies, at least four foetuses from a minimum of 3–5 separate litters were used for the studies described next.

The dose of DBP used for the present studies induces a high incidence of TDS-like disorders and acute and long-term effects on GC development in Wistar rats (Fisher *et al.*, 2003; Ferrara *et al.*, 2006). Animals exposed to DBP in foetal life were killed at various foetal (e14.5, e15.5, e17.5, e19.5, e21.5) or postnatal (postnatal days 6, 8, 10, 15) ages depending on the experiment. To collect foetuses, dams were killed by CO_2_ inhalation followed by cervical dislocation, the foetuses removed, decapitated and stored in ice-cold phosphate-buffered saline (Sigma-Aldrich) prior to microdissection of testes. Postnatal animals under the age of Pnd10 were decapitated and animals over Pnd10 killed by CO_2_ inhalation followed by cervical dislocation prior to testes removal. Testes were either frozen (−80 °C) for RNA analysis or fixed for 30–360 min in Bouin's fixative, depending on age, before being transferred to 70% ethanol. Fixed tissue was processed into paraffin wax blocks using an automated processor.

In animals used to determine the GC proliferation index (PI), 100 mg/kg bromo-2′-deoxyuridine-5′-monophosphate (BrdU; Sigma) was administered in 2 mL/kg saline via intraperitoneal injection to either the pregnant dam (for foetal animals) or directly to the males (postnatal animals) 1.5 h prior to death.

### Immunohistochemical analysis of GC development

Immunohistochemistry was used to identify GCs for enumeration (DAZL (deleted in azoospermia) and VASA) or aggregation (VASA), or to evaluate the progress of GC differentiation [OCT4 (octamer-binding transcription factor 3/4; also termed POU5F1) and DMRT1 (Doublesex and MAB-3-related transcription factor 1)], or to determine the PI using BrdU as a proliferation marker, or to determine GC location within the seminiferous tubules of postnatal animals [smooth muscle actin (SMA) used to label peritubular cells adjacent to the basement membrane]. These specific proteins were detected using specific antibodies (Table 1) and general immunohistochemical methods described and validated elsewhere (Raymond *et al.*, 2000; Fisher *et al.*, 2003; Hutchison *et al.*, 2008). Some antibodies required antigen retrieval for optimal detection (Table 1), by pressure cooking slides for 5 min in 0.01m citrate buffer (pH 6.0). Serum blocking to prevent non-specific background staining used the appropriate normal serum diluted 1 : 5 in Tris-buffered saline (TBS) with 5% bovine serum albumin (Sigma). Slides were incubated overnight with primary antibody (Table 1) at 4 °C and detected using the appropriate secondary antibody conjugated to biotin at 1 : 500 in TBS for 30 min at room temperature. The biotinylated secondary was linked to horseradish peroxidase by 30 min incubation with streptavidin-horseradish peroxide enzyme conjugate (Vector Labs, Peterborough, UK) and visualized by application of diaminobenzidine (liquid DAB; Dako, Glostrup, Denmark). Slides were counterstained with haematoxylin, dehydrated and mounted using Pertex mounting medium (Cell Path, Hemel Hampstead, UK).

**Table 1:** Details of primary antisera used for immunohistochemistry

Antibody	Source	Retrieval	Species	Dilution
BrdU	Fitzgerald Industries, MA	Yes	Sheep	1 : 1000
DAZL	AbD Serotec, Oxford	Yes	Mouse	1 : 300
DMRT1	Gift from David Zarkower	Yes	Rabbit	1 : 2000
OCT4	Santa Cruz, CA, USA	Yes	Goat	1 : 100
SMA	Novocastra, Newcastle	No	Mouse	1 : 1000
VASA	Abcam, Cambridge	Yes	Rabbit	1 : 200

BrdU, bromo-2′-deoxyuridine-5′-monophosphate; DMRT1, Doublesex and MAB-3-related transcription factor 1; OCT4, octamer-binding transcription factor 3/4; SMA, smooth muscle actin.

To determine the GC PI or the percentage of GCs expressing OCT4 or DMRT1, or to determine the position of GCs within the seminiferous cords, double immunohistochemistry was used, essentially as described elsewhere (Fisher *et al.*, 2003). For this, slides underwent second antibody detection for a different antigen after the initial antibody detection. Briefly, after DAB application and TBS washing, slides underwent a second 30-min serum block appropriate to the second primary antibody (Table 1) and the above process was then repeated. To detect the second primary antibody, slides were incubated with the appropriate secondary antibody conjugated to alkaline phosphatase (Dako; 1 : 200) for 30 min at room temperature with visualization using Fast Blue [1 mg Fast Blue salt (Sigma) in 1 mL Fast Blue buffer (12.1 mg Tris, 0.2 mg napthol AS-MX phosphate, 20 μL dimethyl formamide, pH 8.2)]. Slides were then mounted using Permafluor aqueous mounting fluid (Beckham Coulter, High Wycombe, UK). Apoptosis of GCs in complete testis cross-sections at each age, was assessed using TUNEL (terminal deoxynucleotidyl transferase dUTP nick end labelling), as detailed previously (Atanassova *et al.*, 2000). Slides were viewed using a Provis microscope (Olympus Optical AX70, London, UK) and images were captured using a DCS330 digital camera (Eastman Kodak, NY, USA).

### Determination of GC number per testis

GC number per testis was evaluated using DAZL-immunostained slides (Fisher *et al.*, 2003) and stereological methods described previously (Scott *et al.*, 2008; Hutchison *et al.*, 2008). This used Image-Pro 6.2 software (Media Cybernetics UK, Wokingham, Berkshire, UK) and an Olympus BH-2 microscope fitted with a Prior automatic stage (Prior Scientific Instruments Ltd., Cambridge, UK). The number of fields counted per animal was dependent on obtaining a standard error value of ≤5%. Conversion of volume data to cell numbers per testis used the mean nuclear volume of GCs at that age (average of 80–100 nuclei) using the selector function of the software and testis volume (see next). GCs were counted at e14.5, e15.5, e17.5, e19.5, e21.5, Pnd6 and Pnd15 in control and DBP-exposed animals (*n* = 5–8 per group).

Testes from postnatal animals and foetuses aged over e19.5 were weighed directly, but in younger animals, total testis volume was determined by reconstruction from serial sectioning combined with measurements made on photographic images of microdissected gonads as described previously (Scott *et al.*, 2007). Although we have validated this approach (Scott *et al.*, 2007), use of different methods for volume determination at different ages will have introduced different errors, which may also vary according to age. Therefore, we do not consider that comparison of GC numbers at different foetal ages is accurate, whereas any comparison of control and DBP-exposed animals at the same age will be subject to the same errors and are thus comparable.

### Analysis of GC proliferation, differentiation and migration within the seminiferous cords

Two key protein markers were used to evaluate DBP effects on GC differentiation. OCT4 is a pluripotency-associated factor and is the classic marker of CIS/TGCC cells in the human (Rajpert-De Meyts, 2006) and its expression can be altered by foetal DBP exposure (Ferrara *et al.*, 2006). DMRT1 was chosen because (i) it plays a highly conserved role in sexual differentiation (Zarkower, 2001); (ii) it is expressed in foetal GCs and Sertoli cells; (iii) its knockout in mice results in abnormal GC development (Raymond *et al.*, 2000; Kim *et al.*, 2007); (iv) DMRT1 is associated with maintenance of pluripotency and can regulate a number of pluripotency-associated genes, including OCT4 (Krentz *et al.*, 2009; Murphy *et al.*, 2010).

To calculate the GC PI, or the percentage of GCs expressing OCT4 or DMRT1, 30–50 random fields were examined per testis and GCs were counted as positive or negative for the marker in question (BrdU, OCT4 or DMRT1). GC PI was determined at e15.5, e17.5, e19.5, e21.5, Pnd6, Pnd8 and Pnd10. The percentage of OCT4-expressing GCs was determined at e17.5, and the percentage of DMRT1-expressing GCs was determined at e19.5 and Pnd6. The reasons for choice of these ages are explained in the ‘Results’ section.

At Pnd6, the position of GCs in seminiferous cords was investigated using double immunohistochemistry for the GC-specific marker VASA and for SMA (Fisher *et al.*, 2003) to identify peritubular myoid cells and the adjacent basement membrane. GC position was classed as centrally located or basally located, if some of the GC cytoplasm was located at the basement membrane.

### Measurement of GC aggregation at e21.5

DBP exposure causes aggregation of GCs in the foetal testis beyond e19.5 (Fisher *et al.*, 2003; Kleymenova *et al.*, 2005). As all GCs at e21.5 immunostain intensely for VASA, we utilized this to develop a method for objective quantification of aggregation/clustering of GCs using a similar approach to that described for quantification of foetal Leydig cell clustering (Mahood *et al.*, 2005). Sections immunostained for VASA were of sufficient contrast and low background to allow computer-assisted thresholding and quantification by stereological analysis using Image-Pro Plus 4.5.1 with Stereologer-Pro 5 plug-in software (Media Cybernetics UK). Other details are as in Mahood *et al.* (2005). In e21.5 controls, GC clusters varied widely in size (<20–2800 arbitrary units) although most were <1000 units. In DBP-exposed animals, there were fewer but larger GC clusters and ∼15% of cords contained clusters >3000 units, a size rarely found in controls. Various paradigms were explored for discriminating control and DBP-exposed animals, but the most robust was using clusters >3000 units as the cut-off point. Therefore, the percentage of GCs in clusters >3000 units was used as an index of GC aggregation.

### *Oct4* mRNA expression in the foetal testis

To determine *Oct4* mRNA expression levels in whole testes, standard Taqman Q-RT-PCR was used as described elsewhere (Drake *et al.*, 2009), with primers (forward: GAAGTTGGAGAAGGTGGACC; reverse: CCTTCTGCAGGGCTTTCATA) and probe 95 from the Roche universal probe library (http://www.roche-applied-science.com/sis/rtpcr/upl/ezhome.html). Samples (*n* = 6 per age and treatment) were analysed in triplicate (ABI 7900, Applied Biosystems. Carlsbad, California, USA).

### Statistical analysis

Data were analysed using Student's unpaired *t-*test and GraphPad Prism (version 5; GraphPad software Inc., San Diego, CA, USA). Some data were log-transformed prior to statistical analysis to normalize variances. In some instances, more than one animal was used per litter; therefore, to rule out any statistically significant differences as stemming from between-litter, rather than between-treatment, effects, data were also analysed using litter means. This did not alter the results or conclusions, although in some instances, it did reduce the level of statistical significance.

## Results

### Effect of DBP treatment on GC number

**Figure 1:**
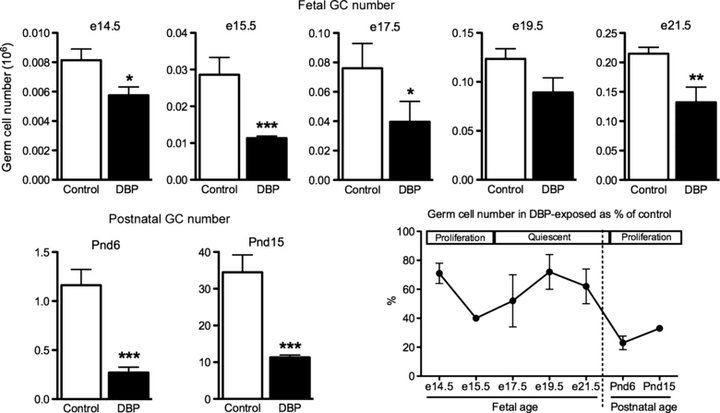
Effect of foetal exposure to di(*n*-butyl) phthalate (DBP; 500 mg/kg/day), on germ-cell (GC) number at various foetal and early postnatal ages in the rat; lower right panel shows the GC number at each age as a percentage of the respective age-matched control in relation to periods of GC proliferative activity. Note that DBP treatment ceased 24 h before the indicated day of foetal sampling or, in the case of postnatal sampling, on e21.5. Values are means ± SEM for 4–10 animals from 4 to 6 litters at each age. *p < 0.05, **p < 0.01, ***p < 0.001, in comparison with the respective control.

At all foetal (e14.5–e21.5) and postnatal (Pnd6, Pnd15) ages investigated, GC number per testis was consistently reduced in DBP-exposed animals, although this reduction just failed to achieve statistical significance at e19.5 (Fig. 1). The magnitude of the DBP-induced reduction increased from e14.5 (29% reduction) to e15.5 (60% reduction), but thereafter, somewhat lesser reductions were found at e17.5 (48% reduction) and at e19.5–e21.5 (28–38%), perhaps reflecting some recovery of GC numbers because of prolongation of GC proliferation in DBP-exposed animals (see next). In contrast, a much more pronounced reduction in GC numbers was found postnatally (77% and 67% reductions at Pnd6 and Pnd15, respectively; Fig. 1), a finding probably also explained by altered GC proliferation on Pnd6 (see next). At face value, our results in controls imply that GC number increases beyond e17.5 when GC proliferation has ceased. However, different methods were used for estimation of testis volume up to e17.5 and thereafter, and this precludes accurate comparison of absolute GC numbers between the different ages (see ‘Materials and methods’).

### Effect of DBP treatment on GC proliferation and apoptosis

**Figure 2:**
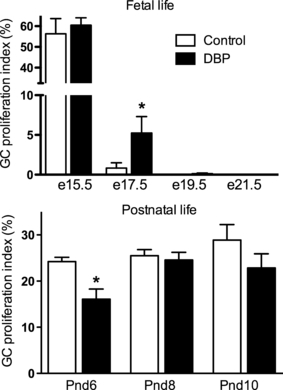
Effect of foetal exposure to di(*n*-butyl) phthalate (DBP; 500 mg/kg/day), commencing at e13.5, on germ-cell (GC) proliferation at various foetal and early postnatal ages in the rat. Note that DBP treatment ceased 24 h before the indicated day of foetal sampling or, in the case of postnatal sampling, on e21.5. Values are means ± SEM for 4–5 animals from four litters at each age. *p < 0.05, in comparison with the respective control.

To determine if increased apoptosis explained the reduction in GC number in DBP-exposed animals, complete testis cross-sections were evaluated systematically, but only occasional apoptotic GCs were observed at e14.5–e17.5 (4–6 per complete testis cross-section) and none at e19.5–e21.5. There was no consistent difference between control and DBP-exposed groups in the numbers of apoptotic GCs (data not shown). We also examined testes from animals 8 h after initial dosing of DBP on e13.5, but at this age, seminiferous cord formation was only initiating and it was thus difficult to identify apoptotic cells (which were rare in any case) unequivocally as GCs. Therefore, although we were unable to demonstrate DBP-induced GC apoptosis soon after treatment on e13.3 or e14.5, this was primarily because of technical/sampling limitations. To determine if reduced proliferation explained the changes in GC number in DBP-exposed animals, the PI (BrdU incorporation) was determined at four foetal and three postnatal ages. In foetal life, GC proliferation was >50% in controls and DBP-exposed animals at e15.5 (Fig. 2). At e17.5, most GCs (>98%) in controls had entered quiescence and this was complete by e19.5, whereas in DBP-exposed animals, significantly more GCs were still proliferating at e17.5 in comparison with controls, although only occasional BrdU-positive cells were evident at e19.5, and none at e21.5 (Fig. 2). This suggested that DBP exposure delayed the entry of some foetal GCs into quiescence.

In contrast, after birth, there was a significant reduction in the GC PI at Pnd6 (proliferation of basally located spermatogonia) in DBP-exposed animals compared with controls, although this effect was not evident at either Pnd8 or Pnd10 (Fig. 2). The reduced proliferation at around Pnd6 will presumably have exacerbated the reduction in numbers of GCs already present from foetal life in DBP-exposed animals, and presumably explains the greater reduction in GC numbers evident in postnatal compared with foetal life (Fig. 1).

### Effect of DBP treatment on GC differentiation

To evaluate other aspects of GC functional differentiation, we chose OCT4, a pluripotency-associated factor and DMRT1, expression of which is important in mouse GC development (Kim *et al.*, 2007).

**Figure 3:**
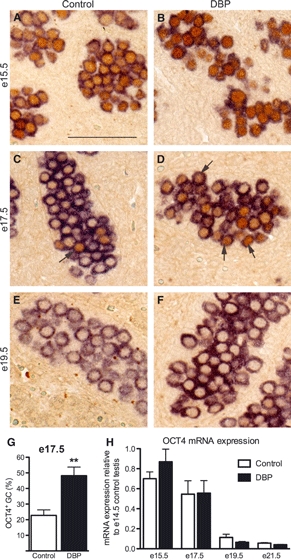
Effect of foetal exposure to di(*n*-butyl) phthalate (DBP; 500 mg/kg/day), commencing at e13.5, on germ-cell (GC) expression of octamer-binding transcription factor 3/4 (OCT4; brown nuclear staining) at e15.5–e19.5 in the rat. GCs are identified by their cytoplasmic staining for VASA (blue/purple colour). Note that DBP treatment ceased 24 h before the indicated day of foetal sampling. Note also that in controls, most GCs immunoexpress OCT4 at e15.5 (A), but this decreases substantially at e17.5 (C,G) and is completely absent at e19.5 (E). DBP exposure increases the percentage of GCs expressing OCT4 at e17.5 (arrows; D,G), but not at e19.5 (F). OCT4 mRNA expression (H) decreased progressively with age and was unaffected by DBP exposure. Values in (G) and (H) are means ± SEM for six animals from 4 to 5 litters at each age. **p < 0.01, in comparison with the respective control.

In controls, most GCs at e15.5 expressed OCT4, declining to ∼20% at e17.5 and to 0 by e19.5 (Fig. 3). In DBP-exposed animals, significantly more GCs (∼50%) expressed OCT4 at e17.5, compared with controls, but by e19.5, most GCs were OCT4-immunonegative (Fig. 3). There was no difference between control and DBP-exposed foetuses in OCT4 mRNA expression, which declined progressively between e15.5 and e21.5 (Fig. 3).

**Figure 4:**
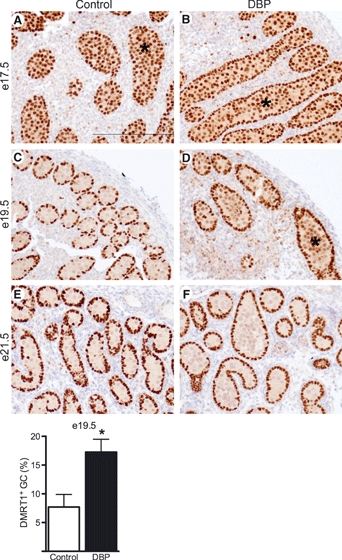
Effect of foetal exposure to di(*n*-butyl) phthalate (DBP; 500 mg/kg/day), commencing at e13.5, on expression of Doublesex and MAB-3-related transcription factor 1 (DMRT1; brown nuclear staining) at e17.5–e21.5 in the rat testis. Immunopositive germ cells (GCs; asterisks) occupy a central position within the seminiferous cords, whereas Sertoli cells, which immunoexpress DMRT1 at all ages, are located in a ring to the periphery. Note that DBP treatment ceased 24 h before the indicated day of foetal sampling. Note also that in controls, most GCs immunoexpress DMRT1 at e17.5 (A), but this decreases to ∼7% at e19.5 (C,G) and to 0 at e21.5 (E). DBP exposure increases the percentage of GCs expressing DMRT1 at e19.5 (D,G) and in only occasional GCs at e21.5 (F). Values in (G) are means ± SEM for four animals per treatment from four separate litters. *p < 0.05, in comparison with the respective control.

**Figure 5:**
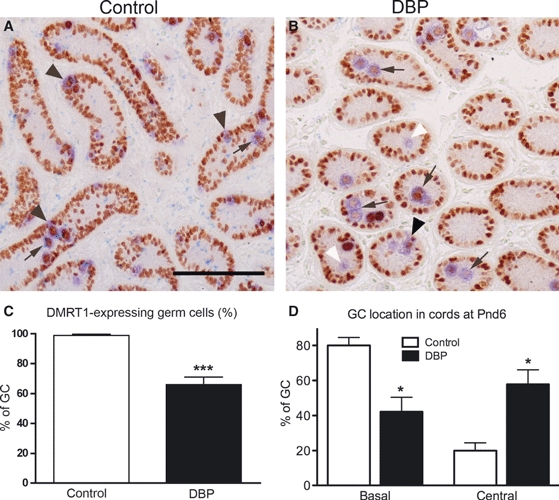
Effect of foetal exposure to di(*n*-butyl) phthalate (DBP; 500 mg/kg/day; e13.5–e21.5) on expression of Doublesex and MAB-3-related transcription factor 1 (DMRT1; brown nuclear staining) on postnatal day 6 in the rat testis. Germ cells (GCs) are identified by their cytoplasmic staining for VASA (blue/purple colour) whereas Sertoli cells, which also immunoexpress DMRT1 do not express VASA. Note that in controls, nearly all GCs immunoexpress DMRT1 (A,C), whereas in DBP-exposed animals, some GCs are negative for DMRT1 [white arrowheads in (B) and quantification in (C)]. In controls, most GCs have migrated to the basement membrane [black arrowheads in (A) and quantification in (D)], whereas in DBP-exposed animals, fewer GCs have completed this migration (B,D). Note that most centrally located GCs are immunopositive for DMRT1 in both controls and DBP-exposed animals (arrows). Values in (C) and (D) are means ± SEM for 4–6 animals per treatment from four litters. *p < 0.05, ***p < 0.001, in comparison with the respective control.

At e17.5 in control and DBP-exposed foetuses, most GCs were immunopositive for DMRT1 (Fig. 4). At e19.5, most (>90%) GCs in controls had switched off DMRT1 expression, which was complete by e21.5. In contrast, significantly more (>15%) GCs in DBP-exposed animals still expressed DMRT1 at e19.5 than in controls, although only occasional DMRT1-immunopositive GCs were still evident by e21.5 (Fig. 4). At all ages, DMRT1 was expressed in all Sertoli cell nuclei, and this was unaffected by DBP treatment. After birth, all GCs remained immunonegative for DMRT1 at Pnd4 (not shown), but by Pnd6 most GCs in controls had switched DMRT1 back on, based on co-expression studies with VASA (Fig. 5A,C), whereas in DBP-exposed animals, only ∼60% of GCs were positive for DMRT1 (Fig. 5B,C). Although there was a reduced migration of GCs to the basement membrane in DBP-exposed animals (Fig. 5D), there was no consistent relationship between absence or presence of DMRT1 immunoexpression and GC position (Fig. 5B).

### DBP effects on GC number and differentiation result from effects early in gestation (e13.5–e15.5)

**Figure 6:**
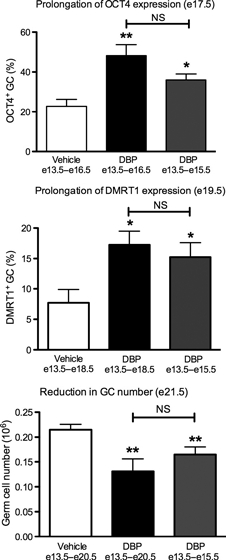
Identification of an early time window (e13.5–e15.5) of foetal exposure to di(*n*-butyl) phthalate (DBP; 500 mg/kg/day) for induction of prolongation of octamer-binding transcription factor 3/4 and Doublesex and MAB-3-related transcription factor 1 immunoexpression in germ cells (GCs) and for induction of a decrease in GC number in the rat. The duration of vehicle/DBP treatment is shown on the *x*-axis of each graph and the age of evaluation in the heading. Values are means ± SEM for 4–10 animals in each treatment group from 4 to 5 litters. *p < 0.05, **p < 0.01, in comparison with the respective control.

Restriction of DBP exposure to the period e13.5–e15.5 induced similar effects to longer period treatment on GC number (e21.5) and prolongation of expression of OCT4 (e17.5) and DMRT1 (e19.5) (Fig. 6).

### DBP exposure in early (e13.5–e15.5) or late (e19.5–e20.5) gestation and GC aggregation at e21.5

**Figure 7:**
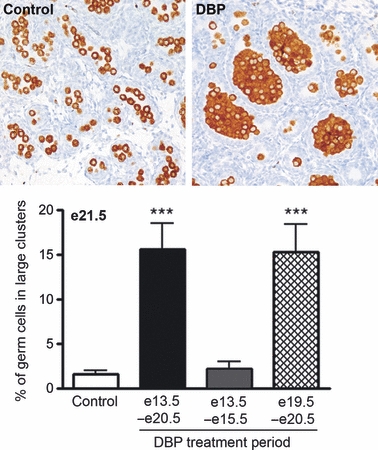
Identification of a late time window (e19.5–e20.5) of foetal exposure to di(*n*-butyl) phthalate (DBP; 500 mg/kg/day) for induction of abnormal germ-cell (GC) aggregation at e21.5 in the rat. GCs were identified by their cytoplasmic staining for VASA (brown colour) and thresholding and image analysis used to compute GC clustering/aggregation (see ‘Materials and methods’). Note that DBP treatment from e13.5 to e15.5 (early window) had no effect. The control group comprises a mixture of animals treated for each of the periods corresponding to those for DBP and were pooled because they did not show any significant treatment-related differences. Values are means ± SEM for 4–10 animals in each treatment group from 4 to 5 litters. ***p < 0.001, in comparison with the control.

DBP-induced GC aggregation becomes evident at e19.5–e21.5 and was assessed at e21.5 in animals exposed in either an early or late time window or throughout the period e13.5–e20.5. This showed that induction of foetal GC aggregation only needed DBP exposure in late gestation and transient exposure in an early time window (e13.5–e15.5) was without subsequent effect (Fig. 7), in contrast to the GC number and differentiation effects (Fig. 6).

## Discussion

These studies have characterized the effects of maternal DBP exposure on GC development in the foetal rat testis, with emphasis on differentiation effects and their time course of induction. Our results show that DBP exposure in the period immediately following testis differentiation in the rat (∼e13.5) causes a major reduction in foetal GC number and delays differentiation of some foetal GCs as manifest by delayed switching off of OCT4 and DMRT1, delayed entry into quiescence, delayed re-emergence from quiescence (after birth) and delayed re-expression of DMRT1. Induction of these effects required DBP exposure only in the period immediately after testis differentiation (e13.5–e15.5), which encompasses the period of gonocyte proliferation and OCT4 expression (Culty, 2009). The 30–50% reduction in GC numbers induced by DBP also stems from this period. In contrast, DBP exposure in this early period did not induce central aggregation of GCs (this study) or of multinucleated gonocytes (Ferrara *et al.*, 2006); these effects only occurred after exposure in a later time window (e19.5–e21.5) when GCs were quiescent and had switched off OCT4 (Ferrara *et al.*, 2006; Culty, 2009). It is presumed that the ‘early’ and ‘late’ GC effects have separate causes.

The present findings confirm and extend previous findings in vivo (Parks *et al.*, 2000; Barlow & Foster, 2003; Fisher *et al.*, 2003; Ferrara *et al.*, 2006; Boekelheide *et al.*, 2009) and in vitro (Chauvigné*et al.*, 2008) in the rat, based on pregnancy exposure to DBP/DEHP and/or their primary metabolites, and are largely consistent with results in vivo (Gaido *et al.*, 2007) and in vitro (Lehraiki *et al.*, 2009) for the mouse. Apoptosis and reduced foetal GC number have also been shown after culturing explants of first trimester human foetal testes for 3 days with 10^−4^m of the DEHP metabolite monoethylhexy phthalate (MEHP; Lambrot *et al.*, 2009). It is therefore of interest that the DBP-induced loss of foetal GCs in the rat occurs similarly early (i.e. immediately after testis differentiation) and is detectable within 24 h of initial exposure (e14.5) to DBP; there was no cumulative effect beyond e15.5 despite continued DBP exposure. Indeed, restricting DBP exposure to e13.5–e15.5 resulted in as big a reduction in GC numbers at e21.5 as did continuous exposure from e13.5 to e20.5. In vitro studies of MEHP effects on foetal mouse testes also showed the greatest reduction in GC numbers after early (e13.5) exposure (Lehraiki *et al.*, 2009). Together, these findings suggest a greater vulnerability of GCs at this early stage to MEHP (or DBP). This finding is relevant to the human, based on in vitro studies (Lambrot *et al.*, 2009), but it remains unknown whether vulnerability to phthalate-induced GC apoptosis would be restricted to the period immediately following testis differentiation (first trimester), as in the human OCT4-immunopositive, proliferating foetal GCs are present throughout gestation, unlike in rodents (Mitchell *et al.*, 2008, 2010). We were unable to show that increased apoptosis explained the DBP-induced reduction in GC number in rats, possibly because of the 8–24 h delay between last DBP exposure and sample collection. However, in vitro studies using rat, mouse and human foetal testis explants have all shown increased GC apoptosis acutely after MEHP exposure (Chauvigné*et al.*, 2008; Lambrot *et al.*, 2009; Lehraiki *et al.*, 2009), so it seems reasonable to conclude that transiently increased apoptosis at around e13.5–e14.5 explains our in vivo findings, especially as DBP clearly did not reduce GC proliferation.

In a previous observational study, we showed that foetal DBP exposure transiently prolonged OCT4 immunoexpression in male, but not in female, GCs (Ferrara *et al.*, 2006). We confirm and quantify this effect in male rats in the present studies and show that it is not because of altered OCT4 mRNA expression. We also show for the first time that there is similar transient prolongation of DMRT1 immunoexpression in foetal GCs in DBP-exposed rats, although this occurred later (e19.5) than for OCT4 (e17.5). Moreover, we show a similar delay in the postnatal switching back on of DMRT1 immunoexpression in some GCs. In contrast, DMRT1 immunoexpression in Sertoli cells was unaltered at any age by DBP exposure. The effects of DBP exposure on DMRT1 immunoexpression are of interest for several reasons. For example, selective loss of GC DMRT1 in mice predisposes to teratoma formation and DMRT1 is a suppressor of downstream gene targets of OCT4, with similar association evidence for human TGCC (Krentz *et al.*, 2009; Murphy *et al.*, in press). The DBP-induced alteration in DMRT1 expression is therefore of potential relevance to the origins/causes of human TGCC, although the pattern of change induced (delayed switching off and on) cannot be related in a straightforward manner to the mouse studies in which it was loss of DMRT1 expression that predisposed to teratoma formation (Krentz *et al.*, 2009).

In mouse GCs after birth, DMRT1 re-expression is reportedly required for normal migration of GCs (differentiating spermatogonia) to the basement membrane (Fahrioglu *et al.*, 2007; Kim *et al.*, 2007). As DBP exposure delayed the postnatal re-expression of DMRT1 in some GCs in the rat as well as reducing the migration of GCs to the basement membrane, the former may explain the latter. Although we have not undertaken a rigorous investigation of this possibility, we noted that DMRT1-immunonegative and -immunopositive GCs were observed in both central and basement membrane locations in Pnd6 rats following foetal DBP exposure.

Arguably, the most important finding from the present studies is the demonstration that all of the effects of DBP exposure on foetal GC number and differentiation were shown to originate within e13.5–e15.5, when GCs are actively proliferating and expressing OCT4 (Ferrara *et al.*, 2006). This was the case irrespective of the age at which a particular effect of DBP exposure was first detectable, namely e14.5 (reduced GC number), e17.5 (OCT4 prolongation) or e19.5 (DMRT1 prolongation). This implies that there are DBP-sensitive mechanisms in GCs during this period, the future identification of which could be important as similar effects on GC number can be induced by MEHP in human foetal testes in vitro at an equivalent stage of development (Lambrot *et al.*, 2009). These might also have relevance to the origins of CIS, especially as they can impact factors such as OCT4 and DMRT1 in GCs (Rajpert-De Meyts & Hoei-Hansen, 2007). Furthermore, this early time window largely predates the ontogeny of Leydig cell steroidogenesis, consistent with evidence from mouse studies that divorce MEHP effects on GCs from either androgen or oestrogen action (Gaido *et al.*, 2007; Lehraiki *et al.*, 2009). In contrast, GC aggregation (also reported as increased seminiferous cord diameter), which first manifests at e19.5–e21.5 in rats (Barlow & Foster, 2003; Kleymenova *et al.*, 2005; Ferrara *et al.*, 2006), was shown to be insensitive to DBP induction by exposure during e13.5–e15.5, but was induced by exposure from e19.5 to e20.5. We showed that DBP induction of multinucleated gonocytes in rats was also restricted to this late time window (Ferrara *et al.*, 2006). This suggests that the ‘early’ and ‘late’ effects of DBP operate through different mechanisms; some studies suggest that the ‘late’ effects are related to altered Sertoli cell function/interaction with the GCs (Kleymenova *et al.*, 2005). No studies have yet reported whether MEHP or other phthalate metabolites can induce foetal GC aggregation or multinucleation in vitro in human foetal testes.

The present study did not explore the dose–response relationship for the GC effects of DBP as the aim was to characterize effects at dose levels of exposure, which induce TDS-like effects in the male offspring. The aim was to identify the period of greatest vulnerability of foetal GC to disruption by phthalate exposure, as this period may prove to be relevant to the human. Whether GC effects, similar to those we describe in rats, would occur in humans at levels of DEHP or DBP exposure reported for the general population (Silva *et al.*, 2004a) is unknown. However, studies comparing levels of primary phthalate metabolites in amniotic fluid in rats after dosing with various doses of DBP with those in normal human pregnancy suggest that target organ exposure in the human foetus may approach those resulting from doses of DBP used to induce effects in rats (Silva *et al.*, 2004b; Calafat *et al.*, 2006). Moreover, studies in rats have shown clearly that there are dose-additive effects of phthalate mixtures (Howdeshell *et al.*, 2008b) and, as humans are exposed to such mixtures (Silva *et al.*, 2004b; Calafat *et al.*, 2006), it is possible that human phthalate exposure may be sufficient to induce foetal GC effects similar to those presently described in the rat.

In conclusion, we show that foetal DBP exposure of the rat induces both numerical and differentiation effects on foetal GCs, which may be relevant to the human, including the origins of CIS/TGCC, either because of when they occur (during period prior to foetal GC differentiation) or because of the endpoints affected (OCT4, DMRT1). As little is known about the regulation of GC development during this period (Culty, 2009), identification of these mechanisms is likely to give new insights relevant to sensitivity of foetal GCs to disrupted development by exposure to phthalates or perhaps to other environmental chemicals, which in turn may give new insights relevant to the origins of CIS. It seems likely from the present findings and those reported in vitro using human foetal testis tissue (Lambrot *et al.*, 2009) that certain phthalates (e.g. DEHP, DBP) have the *potential* to affect adversely foetal GCs in the human testis.
